# The State of Open Source Electronic Health Record Projects: A Software Anthropology Study

**DOI:** 10.2196/medinform.5783

**Published:** 2017-02-24

**Authors:** Mona Alsaffar, Peter Yellowlees, Alberto Odor, Michael Hogarth

**Affiliations:** ^1^ University of California-Davis Department of Health Informatics Sacramento, CA United States; ^2^ University of California-Davis Department of Psychiatry Sacramento, CA United States; ^3^ Health Informatics Graduate Group University of California-Davis Sacramento, CA United States; ^4^ Betty Irene School of Nursing University of California-Davis Sacramento, CA United States; ^5^ University of California-Davis Department of Pathology Sacramento, CA United States

**Keywords:** open source, electronic health record, SourceForge, developers, motivations

## Abstract

**Background:**

Electronic health records (EHR) are a key tool in managing and storing patients’ information. Currently, there are over 50 open source EHR systems available. Functionality and usability are important factors for determining the success of any system. These factors are often a direct reflection of the domain knowledge and developers’ motivations. However, few published studies have focused on the characteristics of free and open source software (F/OSS) EHR systems and none to date have discussed the motivation, knowledge background, and demographic characteristics of the developers involved in open source EHR projects.

**Objective:**

This study analyzed the characteristics of prevailing F/OSS EHR systems and aimed to provide an understanding of the motivation, knowledge background, and characteristics of the developers.

**Methods:**

This study identified F/OSS EHR projects on SourceForge and other websites from May to July 2014. Projects were classified and characterized by license type, downloads, programming languages, spoken languages, project age, development status, supporting materials, top downloads by country, and whether they were “certified” EHRs. Health care F/OSS developers were also surveyed using an online survey.

**Results:**

At the time of the assessment, we uncovered 54 open source EHR projects, but only four of them had been successfully certified under the Office of the National Coordinator for Health Information Technology (ONC Health IT) Certification Program. In the majority of cases, the open source EHR software was downloaded by users in the United States (64.07%, 148,666/232,034), underscoring that there is a significant interest in EHR open source applications in the United States. A survey of EHR open source developers was conducted and a total of 103 developers responded to the online questionnaire. The majority of EHR F/OSS developers (65.3%, 66/101) are participating in F/OSS projects as part of a paid activity and only 25.7% (26/101) of EHR F/OSS developers are, or have been, health care providers in their careers. In addition, 45% (45/99) of developers do not work in the health care field.

**Conclusion:**

The research presented in this study highlights some challenges that may be hindering the future of health care F/OSS. A minority of developers have been health care professionals, and only 55% (54/99) work in the health care field. This undoubtedly limits the ability of functional design of F/OSS EHR systems from being a competitive advantage over prevailing commercial EHR systems. Open source software seems to be a significant interest to many; however, given that only four F/OSS EHR systems are ONC-certified, this interest is unlikely to yield significant adoption of these systems in the United States. Although the Health Information Technology for Economic and Clinical Health (HITECH) act was responsible for a substantial infusion of capital into the EHR marketplace, the lack of a corporate entity in most F/OSS EHR projects translates to a marginal capacity to market the respective F/OSS system and to navigate certification. This likely has further disadvantaged F/OSS EHR adoption in the United States.

## Introduction

### Background

The medical field has been using open source applications for almost 40 years [1]. Electronic health record (EHR) systems first appeared in the early 1960s [2]. The Computer Stored Ambulatory Record (COSTAR) system was the first F/OSS EHR system and was originally developed to be used by the Harvard Community Health Plan. Although COSTAR was implemented in a number of institutions, it did not result in broad national adoption of EHRs at the time. Only the Health Information Technology for Economic and Clinical Health (HITECH) Act of 2009 and its financial incentive program have resulted in broad adoption of EHRs in the United States [3]. F/OSS EHR systems have been increasing in popularity over the period [4].

Although the HITECH incentive payments have increased adoption, EHR adoption continues to have obstacles [5,6]. One of the main obstacles continues to be affordability [5]. CDW Healthcare Physician Practice estimated the total cost of an EHR deployment at approximately USD $120,000 per physician in the first year after implementation, with annual recurring costs of USD $30,000 per physician [7]. Along with the financial cost, there is also the non-financial cost related to time spent to bring the system live and into full functional use [7].

Open source EHR may lessen financial barriers while also providing improved flexibility given that they can be “freely” modified [8]. Many of the prevailing EHRs do not adhere to minimal usability testing standards [9] requiring continuous customization to meet the needs of the organization [10]. A KLAS study of 128 physicians on the current state of acute care EHRs found that no vendor scored high in usability [11]. Since open source software can be freely modified and redistributed, this could reduce the cost of continuous customization to improve usability [12]. Open source projects tend to also benefit from a higher degree of transparency about software anomalies (software bugs), leading to a higher degree of reliability over time. A common belief across the open source community and often referred to as “Linus Law” states “given enough eyeballs, all bugs are shallow” [13]. Unlike organizations who are dependent on a commercial vendor’s prioritization of features and software release schedules, those implementing F/OSS would have complete control over the timing of customizations and deployment, allowing them to choose what functionality is available and when it will be available to their users [14].

F/OSS does come with challenges as well. Although some commercial companies provide support for F/OSS EHRs, the majority of the F/OSS EHR projects do not have a support service one can purchase. This creates a major challenge in ensuring reliability, particularly when the original system has been customized by institutional programmers [15]. Those skeptical of F/OSS EHR systems often highlight the potential dependency on volunteer developers [16] who do not guarantee technical support [15]. In addition, identifying a reliable source for version updates can be challenging [15]. Many organizations also fear that open source projects can become inactive anytime, creating an acute need for substantial in-house software development expertise [17]. A majority of health care organizations do not typically have infrastructure to support software development, and they might not have information technology (IT) staff with expertise in managing the software development lifecycle (SDLC) for complex systems. Instead, the typical health care delivery organization’s IT staff focuses on deploying and optimizing vendor software.

Despite these disadvantages, the F/OSS software has been growing in terms of the number of projects. The 8th Annual Future of Open Source Survey found that the number of F/OSS projects doubled between 2012 [18] and 2014 [19].

The core success of the open source movement depends on developers who contribute their knowledge and effort for free to the community. Developers are either unpaid volunteers, hobbyists [20], or employees who are paid to write code. A study of mainstream F/OSS projects categorized developers’ contribution into eight different roles: project leader, core member, active developers, peripheral developer, bug fixer, bug reporter, reader, and passive user [4]. As reflected in this categorization, there are a number of different roles for contributors and a significant amount of resources required to support a high-quality project. In large part, the developer community and their motivations are a key determinant of success or failure of an open source project. Exploring these motivations is an important aspect of understanding a key success factor for F/OSS EHR systems. The motivation-affecting factors for open source developers can be categorized as internal (cognitive) and external (social). Internal factors are comprised of motivation, altruism, and community identification. External motivation factors include future rewards (eg, peer recognition), self-marketing, human capital, contribution as part of employment (ie, being paid to contribute), and revenue from related products and services [21]. Generally, anything related to the joy of coding is considered intrinsic motivation, whereas extrinsic motivation is associated with receiving some benefit for the contribution. These factors have been explored in mainstream F/OSS projects but have not yet been characterized in F/OSS health care projects.

### Objectives

The objectives of this study were to canvass the current state of open source EHR systems and to characterize the motivations, knowledge, and demographics of the developers.

## Methods

To find EHR F/OSS projects, we used SourceForge, a widely used open source project repository, and Google using the search terms “electronic patient record,” “electronic health record,” “electronic medical records,” and “clinical information system.” The search revealed hundreds of EHR F/OSS projects, but only 54 of them were EHRs according to our study inclusion criteria. The following are two fundamental inclusion criteria used in the study: (1) the software had to be defined as an EHR, such that the project had to adhere to the functional definition of EHR. The HealthIT.gov website defines an EHR as a “digital chart” containing at least the medical and treatment history of the patients; and (2) the software had to use an open source license. Open source software is defined as software without license restrictions on its redistribution and the software can be freely modified. [22].

The study was conducted for a 3-month period starting May 2014. To understand the characteristics of the various EHR projects, we looked at license type, downloads, programming languages, spoken languages, project age, development status, supporting materials, top downloads by country and whether they were “certified” EHRs.

### License Type

Many SourceForge applications are defined by their license type on the application homepage. In this study, the licenses were classified into permissive, restrictive, or highly restrictive [22]. The highly restrictive licenses, such as a general public license (GPL), allow free modification but request that any modification should be contributed back to the community under the same license. Highly restrictive licenses are used more in applications geared toward the end user (eg, games) [23]. License restrictions tend to affect who contributes and accessibility of the source code [23].

### Downloads

We made an assumption that download frequency reflects the popularity of the software. This assumption is a commonly held belief in this research domain [24]. We looked at the download number in the last 12-month period on SourceForge. Around 47 projects (87%, 47/54) in this study have information on downloads, a proxy for use of the software.

### Development Status

Development status shows the readiness of software for day to-day use. This study utilized the SourceForge classification for software readiness. In this study, six software stages were used: planning, pre-alpha, alpha, beta, production, stable, and mature. The software’s status can influence a project’s success and affects the interest of the users and developers [24].

### Project Age

Project age represents the number of years since the project development started. The project’s age, in addition to other factors, is positively related to its ability to attract more users and/or resources, which affect the project’s future sustainability [25].

### Programming Language

Programming language for each software system was examined and classified according to whether one or multiple were used. One open source study suggests that using one common programming language affects the success of the software project [26].

### Spoken Languages

The projects were classified according to their spoken languages. One study proves that open source software popularity is related to the number of language versions available [27].

### Supporting Materials

The setup of the system is not always obvious, and in some cases requires IT administration skills. The top 10 downloaded projects were analyzed, as these materials make the installation and usage of the system easier. The supporting materials included user guides, installation guides, and version demonstrations.

### Top Downloads According to Countries

SourceForge provides important information about the software and gives the highest number of downloads for each EHR software system. The top 10 most downloaded projects were identified, in addition to the country that made the greatest number of downloads, to see if the adoption rate affects the number of downloads.

### Certified Open Source Electronic Health Record

Using a certified EHR is a requirement for payments through the HITECH Act’s EHR-incentive program. Certification of an EHR under the ONC program is an important success factor in the United States. The certified open source EHR products and their specifications from The Centers for Medicare and Medicaid Services (CMS) website were examined (Table 1).

**Table 1:** The Centers for Medicare and Medicaid Services-certified free and open source software electronic health record applications.

Product name	Original practice type	Vender	Product version number	Product classification	Certification year	Certification body
OpenEMR	Ambulatory	OEMR	4.1	Complete EHR^a^	2011	ICSA labs
TolvenEMR	Ambulatory, inpatient	Tolven Inc.	2.1	Complete EHR, modular	2011	ICSA labs
WorldVista	Ambulatory, inpatient	WorldVistA	2.0	N/A	2011	InfoGrad
ClearHealth	Ambulatory	ClearHealth Inc.	3.1.5	Complete EHR	2011	InfoGrad

^a^EHR: electronic health record.

### Survey Data Collection

A survey was conducted in 2014 using the commercial survey tool SurveyGizmo (Boulder, CO). The survey consisted of 20 questions and took approximately 5 minutes to complete (Multimedia Appendix 1). The target audience included anyone who self-identified as a developer of health care F/OSS. Our questionnaire was modeled after a similar survey developed to compare proprietary and open source software in 2003 [28].

An announcement of the survey was published on 10 websites that focused on health care open source news and targeted health care developers. The announcement contained a brief summary of the main goal of the survey along with the author's names and their affiliations. About 1 week after this first announcement, the survey was distributed to 54 open source project developers’ mailing lists obtained from SourceForge. We reached out to project email addresses and asked them to distribute our survey to their mailing lists. In addition, we sent a survey personal invitation to specific developers who mentioned working in F/OSS health care projects in their LinkedIn profile. The survey was posted for 5 weeks and a total of 103 responses were collected.

## Results

### Application Data

The study revealed several key observations. At the time of the study, there were 54 open source EHR projects, but only four had been successfully certified under the Office of the National Coordinator for Health Information Technology (ONC Health IT) Certification Program. Nearly half of the projects (57%, 31/54) used a restrictive license type, and approximately 57% (30/54) used GPL. The data revealed that 52% (28/54) of the projects were in production/stable status, only 2 (4%, 2/54) were in mature status, while 1 (2%, 1/54) project was inactive (Table 2). There were 44 active projects at varying stages of development, while 10 had unspecified status.

**Table 2:** Applications development status (N=54).

Development status	Frequency, n (%)
Production/stable	28 (52%)
Undetermined	11 (20%)
Alpha	6 (11%)
Beta	6 (11%)
Mature	2 (4%)
Pre-alpha	1 (2%)

As one might expect, many open source projects (46%, 25/54) used one programming language. However, a large percentage (36%, 19/54) used multiple programming languages. Approximately 18% (10/54) of the projects did not indicate the use of a specific programming language. The analysis also showed that the number of downloads for the projects that were written using multiple programming languages (n=147,914) were higher than the projects using one programming language (n=115,299). Among those projects indicating a programming language, the “PHP Hypertext Preprocessor”, commonly known as PHP, was the leader, and it was used in 31% (17/54) of the projects.

The OpenEMR project had the highest number of downloads (63,418 in a 12-month period) (Table 3). The data shows that the United States accounts for the majority of the downloads and constitutes 64.07% (148,666/232,034) of the total downloads of open source EHR projects on SourceForge. In total, 19% (10/54) of open source EHR systems have installation and user guides along with demonstration versions. The mean project age is 7 years with a range of 2 to 14 years.

**Table 3:** The characteristics of the top 10 downloaded free and open source software electronic health record systems.

Product name	Installation guide	Demonstration	User guide	Top downloads according to country	Development status	Start year	Age, years
OpenEMR	Yes	Yes	Yes	United States	Production/stable	2002	12
OpenMRS	No	Yes	No	United States	Production/stable	2010	4
Care2x	Yes	Yes	Yes	United States	Production/stable	2002	12
OpenClinic GA	Yes	Yes	Yes	India	Production/stable	2010	4
Open Hospital	Yes	N	Yes	India	Production/stable	2006	8
FreeMED	Yes	Yes	Yes	United States	Production/stable	2000	14
GNU Health	Yes	Yes	Yes	India	Production/stable	2006	8
HOSxP	Yes	Yes	No	Thailand	Production/stable	2002	12
Tolven Health Record	Yes	Yes	Yes	France	Production/stable	2006	8
OSCAR McMaster	Yes	Yes	Yes	Canada	Production/stable	2001	13

In terms of project (spoken) language, a large number of projects were written in English (46%, 25/54), but surprisingly, 28% (15/54) were being developed in one or more languages besides English. It was found that 11% (6/54) of the projects did not specify a language (Table 4). Data shows that the number of downloads for projects with multiple spoken languages was very high (n=187,933); with a download rate three times the one-language projects (n=48,402). These numbers indicate a global interest in F/OSS EHR development and prove that the number of language translations is positively related to project success and popularity.

**Table 4:** Frequency of spoken languages (N=54).

Language	Frequency, n (%)
English	25 (46%)
Non-English	4 (7%)
Multiple (English plus other)	15 (28%)
Multiple (non-English)	4 (7%)
Unspecified	6 (11%)

### Survey Data

A total of 103 developer survey responses were successfully collected showing the developer’s characteristics, background, and their reasons to contribute to health care open source projects. Survey respondents were primarily male (94%, 94/99) with an age range of 25 to 55 years old. The majority of developers were American (37%, 36/98) and 42% (41/98) lived in the United States. Employees made up 58% (58/100) of the sample, and self-employed developers constituted 33.0% (33/100). Approximately 58% (58/99) of developers were married and 18.0% (18/100) had children older than age six. As F/OSS developers tend to be highly educated, many had graduate level (43.0%, 43/100), professional level (18.0%, 18/100), or undergraduate level (36.0%, 36/100) education. Only 3.0% (3/100) of F/OSS developers reported not having formal education beyond high school.

As has been found in mainstream F/OSS projects, the majority of health care F/OSS developers participated in the projects as part of a paid activity. Nearly 34.7% (35/101) of the contributors received direct payment for developing F/OSS. Another 30.7% (31/101) received direct payment for either managing or supporting F/OSS while 34% (32/94) received no payment at all for contributing to the projects. Most of the developers contributed by writing code during their off-work hours (41.6%, 42/101) and (39.6%, 40/101) during their work hours. Almost half of the respondents reported employer awareness of their F/OSS work (42%, 42/99). A number of respondents (32%, 32/99) worked on F/OSS as part of their employment. Some employers (17%, 17/99) were unaware of the developers work on F/OSS, but very few (3%, 3/99) did not want them to contribute to F/OSS development.

Contributors’ efforts have been measured by the number of hours spent on a project per week. Respondents’ answers show an average of 9.8 hours per week spent on F/OSS projects. Health care open source developers were asked how many projects they had contributed to. Nearly 49.0% (50/102) worked on one project while 47% (48/94) had worked on 2 to 5 open source projects. The average number of projects was 1.6 with a maximum number of 5 projects.

A significant number of respondents (47.0%, 47/100) reported 75% to 100% of their code was included in a F/OSS project. Around one third (43.0%, 43/100) reported less than 25% of their code was included in the final project. The majority of contributors (50%, 48/96) wrote between 500 and 5000 code lines, while only 30% (29/96) wrote more than 10,000 lines. The average lines of code were 1776 with a maximum of 5000 lines.

Respondents were asked to answer several questions regarding their F/OSS project to analyze their opinions, motivations, and habits toward F/OSS projects. Interestingly, developers had different motivations to participate in F/OSS. The top reasons for contributing to a health care open source project were based on enjoyment-related intrinsic motivation; the project was “important and visible” (47.5%, 48/101) or “technically interesting” (47.5%, 48/101) for them. Approximately 20.8% (21/101) indicated community-based related intrinsic motivation as their reason to contribute and stated that they knew people working on the project.

The survey results confirmed that many developers start developing F/OSS to give back to the community. Around 40% (39/97) considered it important or very important to give back to the community and 38% (37/96) considered the interaction with like-minded programmers to be important. A significant number of respondents were motivated to promote the mode of development and the ideal of freely modifiable software (47%, 47/99), while the remaining developers were motivated to provide alternatives to proprietary software (53%, 50/93). Some developers (27%, 25/97) began developing F/OSS software by modifying it to fit with their requirements or to fix the bugs in their existing software (32 %, 32/97), and 26% (25/96) of them were interested in learning how the program worked.

## Discussion

### Principal Findings

Open source projects continue being started at a rapid pace, but sustainability of the projects appears to be a challenge. A study in 2005 found 45 F/OSS EHR applications [29] compared to the 54 found in this study. This represents a 16.6% increase in the past 9 years. However, most of the projects are different, suggesting sustainability is a significant challenge. Functional EHR systems are complex with many required subsystems and modules, requiring robust development and change management processes in order to achieve high-quality “production-grade” software for the mission-critical environment of a hospital or clinic. Certification is an added challenge in some markets [30]. Despite these challenges, F/OSS EHR systems have been viewed as excellent options for community health clinics, small practices, and hospitals that are under-capitalized in the United States and overseas [8].

In terms of open source licenses, the majority F/OSS projects use GPL, which allows free modification but requires that any modification be contributed back to that open source project under the same license. This creates a “poison pill,” making it difficult for commercial entities to integrate GPL open source components into a module that also has proprietary software. This can be viewed as a “restriction” with regards to open source licensing. Restrictiveness of the license also affects a number of contributors and accessibility of the source code [23].

With regards to developers, this study found the average F/OSS contributor is well-educated, young, and male; the F/OSS community is male dominant, as women contributors are only 2%. The reasons for gender inequities remain unclear but may involve women facing hybrid discriminations from a F/OSS community [31]. Our research shows marital status and having kids older than 6 years old increases the probability a person will volunteer [32,33].

One of the most common aspects of F/OSS is that they are based on voluntary efforts of developers. The goal of this research was to understand what motivates these developers to contribute to a health care F/OSS project. Learning new skills and becoming a better programmer is one of the motivations this research found (35%, 34/96), which is similar to previous findings about mainstream F/OSS developers (36.5%) [28]. Learning and acquiring new skills appears to be another important motivation for many developers.

Intrinsic motivations, such as altruism, are high among health care developers; 47.5% (48/101) of developers worked on the programs for this reason. Only 16% of developers in other research papers stated that altruism was their prime motivator [21]. Contrary to our research findings, some previous research papers [34,35] found that only a minority were motivated by extrinsic motivation, which involved payments for their participation in F/OSS projects.

One of the unique findings in this research is that 74.2% (75/101) of health care F/OSS developers are not health care practitioners, and 45% (45/99) do not work in the health care field. Being a developer outside of the health care field can be a core problem for the development of usable clinical software with a high degree of functionality. This may serve to explain why open source EHRs have limited functionality today.

Although the open source projects solve licensing cost problems, there is a need for maintenance and implementation costs, which can be a barrier for organizations without health IT expertise. There are many open source EHR options on SourceForge, but not all of them can fit in the clinical workflow in the United States or in certain hospitals.

### Challenges

The availability of motivated developers and the need to continuously improve EHR systems will likely mean F/OSS EHR will continue to be part of the health care software landscape despite the many challenges these projects face today. Characterizing the challenges and benefits of adopting open source EHR will be important in understanding the value of these systems.

### Usability

This study shows a potential weakness for F/OSS EHR projects stemming from developer background, and their ability to understand some of the nuances in health care workflows. A large fraction of developers (74.2%, 75/101) were not health care practitioners and few developers (54%, 54/99) had worked in the health care field. A potential solution to this issue would be to have programs that give F/OSS EHR developers direct access to providers and care venues.

### Interoperability

Many hospitals would benefit greatly from integrated software, rather than a disparate group of systems. However, the F/OSS option can be difficult to interface with commercial systems and may require personnel with multiple skill sets [36]

### Privacy and Security

Maintaining patient privacy through robust security is a critical aspect of any EHR system. A study of F/OSS health care systems found the information is often not safeguarded with consent or privacy policies and offers limited protection against unauthorized access or release of information [37]. However, we did find one open source EHR system (Tolven eCHR), which supported encryption of health care data at the row level in a relational database. This would make data unreadable even to the database administrator of the system. This is a high degree of security and not typical among EHR systems at the time.

### Lack of Financial and Professional Expertise

Low acquisition, installation, and ownership costs make the F/OSS system an excellent option for organizations with limited capital. However, in general, the health care environments that could benefit the most from F/OSS EHR systems tend to have low information technology capital budgets and very limited access to health informatics professionals. These challenges may prevent the successful implementation of any health IT technology, including F/OSS EHR [36]. Our study shows that developing countries adopted F/OSS EHR systems but the major adoptions were in North America after the HITECH act. This suggests that even F/OSS EHR system implementations require substantial financial and workforce resource capacity to succeed.

### Cost

Although F/OSS software does not have licensing costs, effective implementation still requires skilled staff, time for installation, and time for learning the software. F/OSS EHR implementation cost can be as high as the proprietary software because of the add-ons, consultation costs, and need for assistance [36]. An important aspect of the total cost of ownership of an EHR system is usability. Poor usability can directly impact the productivity of expensive health care providers and support personnel. The ability to freely modify F/OSS EHR systems and optimize them for local use likely translates into lower overall cost.

### Skilled Information Technology Personnel

Adopting F/OSS EHR requires a large number of IT employees with specific programming skills who understand the program well. A hospital that chooses to use F/OSS EHR will need to hire developer IT staff and contract with extra IT vendors or consultant support [36].

### Limited Functionality of Clinical Decision Support

At an operational level, F/OSS software also presented reduced functions in decision support and knowledge management. Clinical reasoning, guidelines and protocols, quality assurance, and integrated care were rather limited or nonexistent in most applications [37]. Developers lack medical knowledge as the majority of them are not health care practitioners, which may affect the efficacy of clinical decision support functionality.

### Lack of Liability and Accountability

One of the major shortcomings is the lack of liability and accountability in F/OSS. Our study did not address this issue. Few studies of F/OSS in health care have addressed the risks of using F/OSS. F/OSS EHR projects come without warranties regarding the development, release date, or fulfillment of functionality. F/OSS EHR systems do not have a commercial entity providing support and tend to rely on a volunteer community. This can be a significant risk for a software system that supports the core business of a hospital [36].

### Scope and Limitations

Data on F/OSS EHR projects were primarily collected from SourceForge. Therefore, this study is a snapshot in time, with projects being added and deleted before or after data collection. This amount of “churn” reflects a dynamic software category where our findings may not apply in the future.

Not all F/OSS EHR projects were listed on SourceForge; some projects are managed through independent websites. Therefore, some of the projects on SourceForge were outdated, may have contained inaccurate information, and were a small representation of F/OSS EHR projects as a whole. SourceForge was the main source for most of the data because it tends to be one of the preferred project management platforms for the open source movement and has numerous projects and registered developers.

Survey questions were mostly closed-ended which restricted respondents’ answer options. However, we did accommodate all possible answers by providing an additional “other” option for some questions. There is a possibility that respondents misunderstood questions as there was no usability testing done on the survey prior to using it in the general F/OSS EHR community; however, we did not receive any respondent requests to clarify any questions. Furthermore, the survey respondents do not reflect the entire population of open source developers, but we believe it is a reasonable representative of the target population.

### Conclusions

This study highlights a number of important aspects of F/OSS EHR. Open source software systems seem to be important to some health care organizations; however, only four F/OSS EHR systems are ONC-certified in the United States, which creates a barrier to broader use. Health care open source software also currently lacks directed corporate or governmental support for sustainability and growth of these software programs. We hope this research underlines the challenges that hinder the future of F/OSS and provides avenues for future research to study and improve adoption of F/OSS systems in the United States.

## Appendix

Multimedia Appendix 1

## Abbreviations

COSTAR: Computer Stored Ambulatory Record
EHR: electronic health record
F/OSS: free and open source software
GPL: general public license
HITECH: Health Information Technology for Economic and Clinical Health
IT: information technology
ONC: Office of the National Coordinator
