# Supplementary material for: The State of Open Source Electronic Health Record Projects: A Software Anthropology Study
Source: JMIR Med Inform. 2017 Feb 24;5(1):e6. doi: 10.2196/medinform.5783 (PMC5346165; doi:10.2196/medinform.5783)
Supplement: Multimedia Appendix 1 [file medinform_v5i1e6_app1.pdf]

Survey Questions

1. How many Healthcare F/OSS projects have you contributed to?

- 1
- 2-5
- 5-8
- More than 10

2. Why did you choose to participate in Health Care F/OSS project? (check whichever one is currently most applicable)

- It was an important and visible project
- It seemed technically interesting
- I knew people who were working on it
- The software being developed would be useful to me
- I launched the project
- The company (Healthcare organization) that I worked for uses the software
- Other please specify: Please enter an 'other' value for this selection.

3. Approximately what is the average number of hours per week you contributed to Health Care F/OSS project?

less than 5
5-10
10-15
15-20
20-25
greater than30

4. What percentage of the code you've submitted has been included in the project's release version?

- Less than 25%
- 25-50%
- 50-75%
- 75-100%

5. How important were the following factors when you first started developing Healthcare F/OSS?

5. How important were the following factors when you first started developing Healthcare F/OSS?	Very important	Important	A bit important	Not important
a) I thought it was the best way for software to be developed	○	○	○	○
b) I thought we should all be free to modify the software we use	○	○	○	○
c) I needed to perform tasks that could only be done with modified versions of existing software	○	○	○	○
d) I needed to fix bugs in existing software	○	○	○	○
e) As a user of free software, I wanted to give something back to the community	○	○	○	○
f) I wanted to help provide alternatives to proprietary software	○	○	○	○
g) I wanted to interact with like-minded programmers	○	○	○	○
h) I saw it as a way to become a better programmer	○	○	○	○
i) I liked the challenge of fixing bugs and problems in existing software	○	○	○	○
j) I wanted to find out more about how a particular program worked	○	○	○	○
k) My employer wanted me to collaborate in open source development	○	○	○	○

6. Approximately how many lines of code have you contributed?

- ◉ 500-5000
- ○ 5000-10000
- ○ More than 10000

7. Which of the following is the most appropriate description of your present occupational status?

- Student
- Employee
- Self-employed
- Not employed

8. Which of the following statements are true for you? (check whichever one is currently most applicable)

☐ I am employed by healthcare organization and I get paid for my participation in F/OSS development

☐ My school/employer knows that I work on F/OSS

☐ My school/employer has assigned me to work on F/OSS on the job

☐ My school/employer is unaware of my involvement in F/OSS

☐ My school/employer does not want me to collaborate in F/OSS development

☐ I do not currently have an employer

9. Do you earn money from Healthcare F/OSS, either directly or indirectly? (check whichever one is currently most applicable)

☐ No

☐ Yes, directly: I am paid for developing F/OSS

☐ Yes, directly: I am paid for supporting F/OSS

☐ Yes, directly: I am paid for managing F/OSS

☐ Yes, indirectly: I got my job because of my previous F/OSS experience

☐ Yes, indirectly: My job description does not include F/OSS development

☐ Yes, indirectly: But I also develop F/OSS in my work

☐ Yes, [illegible]ther reasons - Please specify: Please enter an 'other' value for this selection. ____

10. When do you work on open source/free software projects? (check whichever one is currently most applicable)

☐ Before I go to work

☐ At work, during work hours

☐ Off work hours

☐ On weekends

- [ ] I'm not employed right now, so whenever I want

11. What is your gender?

Male
Female

12. What is your marital status/domestic situation? (check whichever one is currently most applicable)

- [ ] Unmarried, without partner
- [ ] Unmarried, not living with partner
- [ ] Unmarried, living with partner
- [ ] Married, not living with spouse
- [ ] Married, living with spouse
- [ ] Separated/Divorced

13. What is your highest formal education level?

High school
Undergraduate
Graduate (Master's or Doctoral)
Professional (JD, MBA, etc.)

14. Have you ever worked in the healthcare ?

Yes
No

15. Are you a healthcare practitioner (physician, nurse, pharmacist ...etc.)?

- ( ) Yes
- ( ) No
- ( ) If yes, please specify Please enter an 'other' value for this selection. [ ]

16. What is your nationality?

[▼]

17. What country do you live in?

[▼]

18. Which one of these free/ open source software have you contributed to?

- [ ] OpenEMR
- [ ] OpenMRS

- Care2X
- Open Clinic Ga
- FreeMED
- Open Hospital
- GNU health
- HOSxP
- Hospital Management System
- Tolven Health Record
- CottageMed EHR
- WorldVista EHR
- OpenPatientOS
- e-Svaasthya
- OSCAR McMaster

19. Do you have children?

- No
- Yes, younger than age 6
- Yes, older than age 6

20. Which age range do you fall into?

- less than 25
- 25-35
- 35-45
- 45-55
- 55-65
- 65-75
- more than 75
